# Physiological Response of Nutrient-Stressed *Lemna gibba* to Pulse Colloidal Silver Treatment

**DOI:** 10.3390/plants12061367

**Published:** 2023-03-19

**Authors:** Martina Varga, Tanja Žuna Pfeiffer, Lidija Begović, Selma Mlinarić, Janja Horvatić, Tihana Miloloža, Ivna Štolfa Čamagajevac

**Affiliations:** Department of Biology, Josip Juraj Strossmayer University of Osijek, Ulica Cara Hadrijana 8/A, HR-31000 Osijek, Croatia

**Keywords:** colloidal silver, short-term exposure, *Lemna gibba*, nutrient-stressed, oxidative stress

## Abstract

Wastewater is a source of many environmental pollutants and potentially high concentrations of essential plant nutrients. Site-specific nutrient levels may influence the response of exposed plants to a chemical stressor. In the present study, we focused on the responses of model aquatic macrophyte swollen duckweed (*Lemna gibba* L.) to a short pulse exposure and a commercially available colloidal silver product as a potential environmental chemical stressor, combined with two levels of total nitrogen and phosphorus nutrition. Treatment with the commercially available colloidal silver product caused oxidative stress in *L. gibba* plants under both high and low nutrient levels. Plants grown and treated under high nutrient levels showed lower levels of lipid peroxidation and hydrogen peroxide accumulation, as well as higher levels of photosynthetic pigment content in comparison to treated plants under low nutrient levels. Higher free radical scavenging activity for plants treated with silver in combination with high nutrient levels resulted in better overall protection from silver-induced oxidative stress. The results showed that external nutrient levels significantly affected the *L. gibba* plant’s response to the colloidal silver presence in the environment and that nutrient levels should be considered in the assessment of potential environmental impact for contaminants.

## 1. Introduction

Natural water bodies receive numerous contaminants through domestic, industrial, and agricultural wastewater, and water pollution is one of the major problems of the present time [1,2]. These pollutants include heavy metals and radioactive elements, industrial solvents, products of fuel combustion, pesticides, fertilizers, and other agrochemicals, detergents, cleaners, paints, and other household products, microplastics, nanoparticles, personal care products, antibiotics, and other human and veterinarian medicines [3,4]. In addition, effluent discharge from wastewater treatment plants and runoff from livestock and crop landscapes result in the presence of nutrient stressors in receiving surface waters [5].

Nanoparticles are contaminants of emerging concern due to a surge in production, especially regarding commercially available products containing silver nanoparticles (AgNPs) known for their antibacterial activity. One category of silver-containing products with a reported increase in production and consumption in recent years is colloidal silver, commercially available as sanitizing sprays, immune boosters, and dietary supplements. Since colloidal silver is defined as a suspension of silver-containing particles with sizes ranging from 1 to 1000 nm [6], a high degree of variability in component formulations and concentrations is expected and reported [6,7,8,9]. AgNPs are inevitably introduced into aquatic environments during their production, storage, and considerable use in everyday life [10]. It has been reported that concentrations of AgNPs in wastewater treatment plants from Europe and the USA are up to 1–5.8 mg kg^−1^ [11]. There are various transformation pathways for AgNPs in the aquatic environment: aggregation, dissolution, sulfidation, chlorination, the release of silver ions from the NPs, and photoreduction of Ag^+^ to the nanoparticle form [10,12], as well as different types of biological transformations [13]. In addition to sedimentation and accumulation by different aquatic organisms, transformation processes may result in a rapid reduction in silver concentration loaded into the aquatic environment through wastewater discharge. Thus, aquatic organisms could be exposed to high silver concentrations for a short time through pulse exposure events. Many studies have focused on the pesticide pulse exposures on aquatic primary producers with a broad range of reported effects [14,15,16,17]; however, reports for short-term metal exposures are scarce [17,18,19]. In addition, very few research studies have focused on the interaction effects of pollutants and nutrient exposure levels in freshwater macrophytes. Nutrient exposure levels have influenced the dose–response growth curves for *L. gibba* exposed to triclosan [5], and *Lemna minor* exposed to copper [20]. Meanwhile, increasing nitrogen and phosphorus supplies have enhanced *Lemna aequinoctialis* tolerance to cadmium [21], as well as *L. minor* and *Spirodela polyrhiza* tolerance to lead [22]. Yu et al. [23] also found that high nutrient levels could alleviate the impact of high-dose microplastic exposure to the aquatic carnivorous plant *Utricularia vulgaris*. Therefore, high nutrient levels can effectively compensate for the low growth rates of plants under stress [23]. Duckweed species are suitable model plants due to their high ecological significance as a primary producer and their role in aquatic environments as a source of food and habitat for many different animals [24], as well as their high sensitivity to numerous potential pollutants [25]. Additionally, Rozman et al. [26] found that external environmental conditions may affect the abiotic stress response mechanisms of treated duckweed.

Previously, we reported that a commercially available colloidal-silver-containing product caused concentration-dependent growth reduction and different physiological changes in treated duckweed plants [27]. Therefore, in this study, we focused on the effects of total nitrogen and total phosphorus nutrition on the ability of *L. gibba* to adequately respond to short-term pulse exposure to a colloidal-silver-containing commercially available product as a potential exposure scenario in the environment. Cellular damage upon exposure to silver could be induced by oxidative stress through an increase in ROS, such as superoxide anions, hydrogen peroxide, and hydroxyl radicals [28]. The accumulation of hydrogen peroxide and lipid peroxidation end-products was assessed to explore the potential of the colloidal silver product to induce oxidative stress when exposure was combined with different nutrient conditions. Additionally, the antioxidative response of *L. gibba* was analyzed through changes in the content of ascorbic acid and soluble phenolic compound concentration, and the activity of the antioxidant enzymes superoxide dismutase, catalase, guaiacol peroxidase, ascorbate peroxidase, glutathione reductase, and glutathione S-transferase. This study aims to contribute to a better understanding of the effects of nutrients on the physiological responses of plants undergoing short-term metal exposure.

## 2. Results

### 2.1. The Effects of Total Nitrogen and Phosphorus Nutrition on Duckweed’s Growth Rate

**Figure 1:**
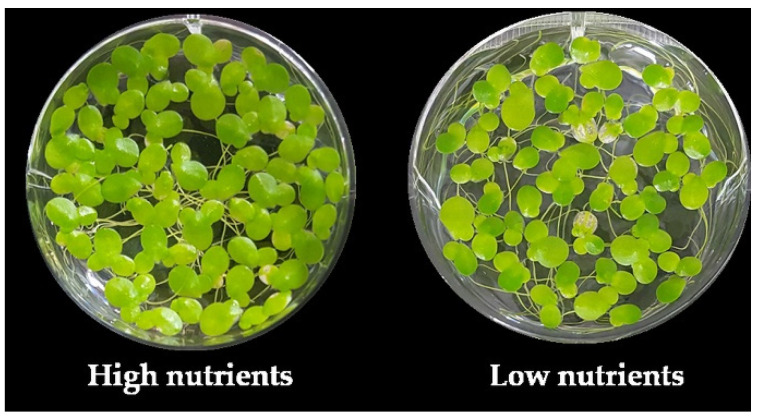
*L. gibba* plants grown under high and low total nitrogen and total phosphorus concentrations in nutrient solution.

Prior to colloidal silver pulse exposure, duckweed plants were cultivated in high nutrient and low nutrient conditions (Figure 1). Plants grown under higher nutrient levels had significantly higher frond number growth rates, a lower corresponding doubling time, and higher total fresh weight (Table 1). Frond number and fresh weight growth rates were 38% and 36% higher for plants grown in high nutrients compared with low-nutrient-grown plants, respectively.

### 2.2. Photosynthetic Pigment Content

**Figure 2:**
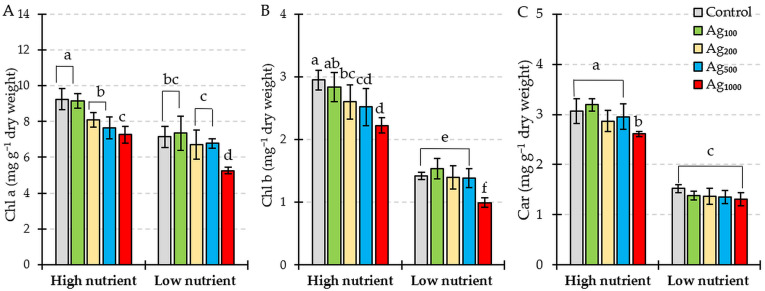
(**A**) Chlorophyll *a* (Chl *a*), (**B**) chlorophyll *b* (Chl *b*), and (**C**) carotenoid (Car) concentration in *L. gibba* exposed to a two-hour pulse of different colloidal silver concentrations (control = 0 µg/L; Ag_100_ = 100 µg/L; Ag_200_ = 200 µg/L; Ag_500_ = 500 µg/L; Ag_1000_ = 1000 µg/L) combined with two levels of total nitrogen and total phosphorus nutrition (high nutrients; low nutrients). Values shown are mean ± standard deviation of ten replicates (*n* = 10). Different letters indicate significantly different values (HSD, *p* < 0.05).

Different nutrient levels interfered with chlorophyll *a*, chlorophyll *b*, and carotenoid content in *L. gibba* plants (Figure 2). In the controls (0 µg/L silver treatment), all measured photosynthetic pigment concentrations were significantly lower under low nutrient conditions. Two-hour colloidal silver pulse exposure in concentrations higher than 200 µg/L significantly reduced chlorophyll *a* (Figure 2A) and chlorophyll *b* (Figure 2B) content in *L. gibba* plants under high nutrient conditions. Under low nutrient levels, only the highest silver concentration caused a significant reduction in chlorophyll *a* and chlorophyll *b* content in comparison to the respective control.

Carotenoid concentration was significantly lower in plants treated with low nutrient conditions compared to high nutrient levels, and no significant differences were observed when low-nutrient-grown plants were exposed to colloidal silver pulse treatment (Figure 2C). There were no significant differences in the carotenoid content of plants exposed to the pulse silver treatment in the concentration range of 100–500 µg/L combined with high nutrient conditions. However, pulse exposure to 1000 µg/L colloidal silver under high nutrient conditions significantly reduced carotenoid content compared to the respective control (Figure 2C).

### 2.3. Biomarkers of Oxidative Stress

**Figure 3:**
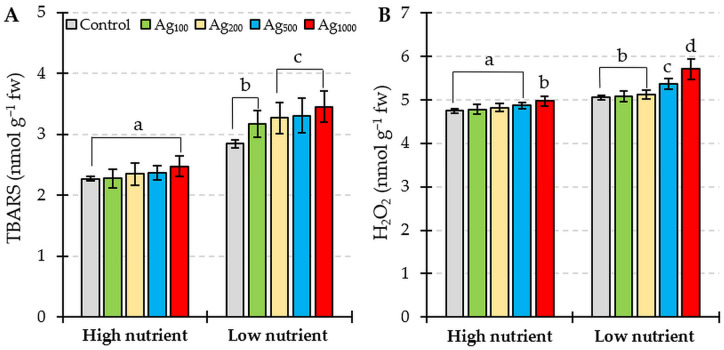
(**A**) TBARS and (**B**) H_2_O_2_ concentration in *L. gibba* exposed to a two-hour pulse of different colloidal silver concentrations (control = 0 µg/L; Ag_100_ = 100 µg/L; Ag_200_ = 200 µg/L; Ag_500_ = 500 µg/L; Ag_1000_ = 1000 µg/L) combined with two levels of total nitrogen and total phosphorus nutrition (high nutrients; low nutrients). Values shown are mean ± standard deviation of ten replicates (*n* = 10). Different letters indicate significantly different values (HSD, *p* < 0.05).

The lipid peroxidation process in *L. gibba* plants was affected by both nutrient levels (F = 309.89, *p* < 0.01) and silver treatment (F = 7.67, *p* < 0.01). TBARS concentration was 25% higher in control plants (0 µg/L Ag) under low nutrient levels compared to plants cultivated under high nutrient levels. Significantly higher TBARS concentrations were found in the combined treatment under low nutrient levels and all colloidal silver concentrations. In contrast, no significant effect of the silver pulse was found in treatments with all silver concentrations being under high nutrient levels (Figure 3A).

Similarly, the H_2_O_2_ concentration in *L. gibba* was affected by both tested factors (nutrient levels: F = 268.60, *p* < 0.01; silver treatment F = 38.24, *p* < 0.01). Significantly higher values were recorded for control plants under the low nutrient conditions than control plants under high nutrient levels (Figure 3B). When the silver pulse treatment was combined with high nutrient levels, no significant differences in H_2_O_2_ concentrations were found in the concentration range of 100–500 µg/L, while 1000 µg/L of silver pulse significantly increased the H_2_O_2_ values compared to the respected control. Under low nutrient conditions, significantly higher H_2_O_2_ values were found in pulse treatments with 500 and 1000 µg/L compared to the control plants. Significantly higher values for H_2_O_2_ concentration were found in plants treated with all of the tested colloidal silver concentrations under low nutrient levels compared to the same silver concentration under high nutrient levels.

### 2.4. Antioxidative Response

#### 2.4.1. Antioxidative Enzyme Activities

**Figure 4:**
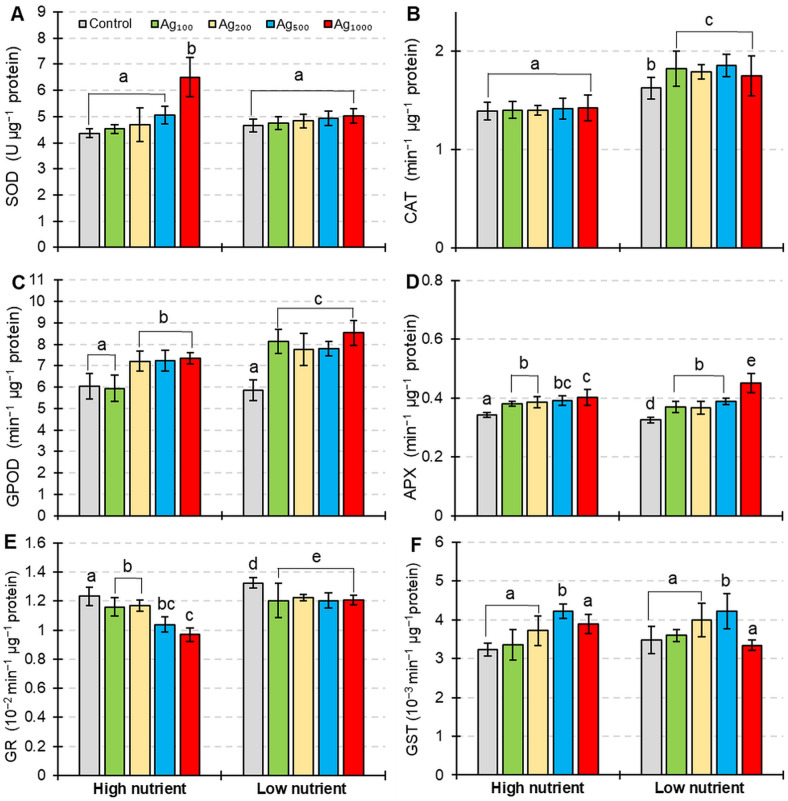
The specific activity of (**A**) SOD, (**B**) CAT, (**C**) GPOD, (**D**) APX, (**E**) GR, and (**F**) GST in *L. gibba* exposed to a two-hour pulse of different colloidal silver concentrations (control = 0 µg/L; Ag_100_ = 100 µg/L; Ag_200_ = 200 µg/L; Ag_500_ = 500 µg/L; Ag_1000_ = 1000 µg/L) combined with two levels of total nitrogen and total phosphorus nutrition (high nutrients; low nutrients). Values shown are mean ± standard deviation of ten replicates (*n* = 10). Different letters indicate significantly different values (HSD, *p* < 0.05).

There was no significant effect of nutrient levels on the SOD activity in *L. gibba* plants before the silver pulse treatment (Figure 4A). While silver treatment had no significant effect on SOD activity in plants under low nutrient conditions, under high nutrient concentration, the silver pulse treatment at a concentration of 1000 µg/L caused significantly higher SOD activity compared to the respective control.

Control plants (0 µg/L colloid silver treatment) grown in low nutrient conditions had significantly higher CAT activity compared to the plants cultivated under high nutrient levels (Figure 4B). Significantly higher levels of CAT activity were recorded in all silver treatments under low nutrient conditions, while the silver pulse treatment caused no change in CAT activity under high nutrient levels.

Different nutrient levels had no significant effect on the GPOD activity in *L. gibba* plants prior to the silver pulse treatment (Figure 4C). When the silver pulse treatment was combined with high nutrient levels, significantly higher GPOD activity was measured in treatments with a silver concentration range of 200–1000 µg/L. Under low nutrient conditions, all colloidal silver pulse treatments caused significantly higher GPOD activity compared to the respective control.

On the other hand, APX activity was influenced by both nutrient levels and silver pulse treatment (Figure 4D). Higher APX activity was measured in plants grown under high nutrient conditions. Silver pulse caused an increase in APX activity similarly for both high and low nutrient conditions. The only exception was the treatment with the highest silver concentration when APX was higher under low nutrient conditions compared to the same treatment under high nutrient levels.

Contrary to APX, the GR activity was higher in plants grown under low nutrient levels (Figure 4E), and all silver pulse treatments caused a significant reduction in GR activity under both high and low nutrient conditions. This reduction was more pronounced when silver treatment was in combination with the high nutrient conditions.

The silver pulse treatment caused an increase in GST activity in plants under both tested nutrient levels (Figure 4F) with significantly higher activity in pulse treatment with 500 µg/L of colloidal silver. When plants were treated with the highest silver concentration, there was no significant difference in GST activity under both high and low nutrient levels.

#### 2.4.2. Non-Enzymatic Antioxidants and Free Radical Scavenging Activity

**Figure 5:**
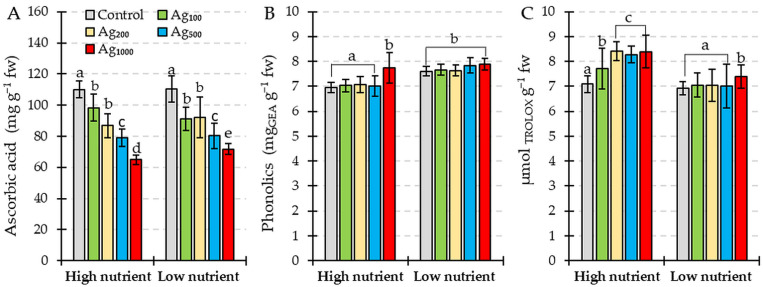
(**A**) Ascorbic acid, (**B**) total soluble phenolic compound concentration, and (**C**) free radical scavenging activity of *L. gibba* exposed to a two-hour pulse of different colloidal silver concentrations (control = 0 µg/L; Ag_100_ = 100 µg/L; Ag_200_ = 200 µg/L; Ag_500_ = 500 µg/L; Ag_1000_ = 1000 µg/L) combined with two levels of total nitrogen and total phosphorus nutrition (high nutrients; low nutrients). Values shown are mean ± standard deviation of ten replicates (*n* = 10). Different letters indicate significantly different values (HSD, *p* < 0.05).

There was no significant effect of total nitrogen and phosphorus nutrition on the ascorbic acid content in *L. gibba* plants before the two-hour silver pulse (Figure 5A). On average, the ascorbic acid concentration in control plants under both high and low nutrient levels was 110.16 ± 6.8 mg g^−1^ fresh weight. The silver pulse treatment caused a concentration-dependent reduction in ascorbic acid concentration similarly for both high and low nutrient conditions, except in the pulse treatment with the highest silver concentration. The ascorbic acid concentration was significantly lower in the 1000 µg/L silver pulse treatment combined with a high nutrient level (64.94 ± 3.01 mg ascorbic acid g^−1^ fresh weight) when compared with the same silver pulse treatment under a low nutrient level (71.77 ± 3.68 mg ascorbic acid g^−1^ fresh weight).

Different nutrient levels interfered with the total soluble phenolic compounds’ concentration (F = 49.32; *p* < 0.01), with significantly higher values found in plants grown under low nutrient conditions (Figure 5B). There was no statistically significant effect of the silver pulse treatment under low nutrient conditions compared to the respective control. Under high nutrient conditions, the highest silver pulse concentration caused a significant increase in total soluble phenolic compound concentration compared to the respective control.

Free radical scavenging activity was generally higher in plants grown under high nutrient levels (Figure 5C). Under high nutrient levels, all silver pulse treatments resulted in significantly higher free radical scavenging activity compared to the respective control. When the silver pulse was combined with low nutrient levels, only the highest silver concentration caused significantly higher free radical scavenging activity compared to the respective control.

## 3. Discussion

The site-specific impact of contaminants is a well-recognized issue in aquatic ecotoxicology [5,29]. Aquatic organisms are routinely exposed to different contaminants and nutrients in complex mixtures in environments influenced by industrial, urban, and agricultural wastewater [5]. Therefore, site-specific nutrient levels may influence the response of exposed plants to a chemical stressor. In the present study, we examined the responses of model aquatic macrophyte *L. gibba* to a short pulse of a commercially available colloidal silver product as a potential environmental chemical stressor, combined with two levels of total N and P nutrition.

*L. gibba* growth rates were clearly influenced by total N and P concentration, with significantly lower growth rates in low nutrient conditions. Fulton et al. [5] also showed that macrophyte growth is a function of N and P concentration in exposure media. The growth of *L. gibba* may be negatively influenced by reduced levels of photosynthetic pigments found in plants cultivated in low nutrient conditions (Figure 2). Moreover, the concentrations of Chl *a* and Chl *b* decreased significantly after two-hour pulse exposure to colloidal silver in both the high and low nutrient conditions, with significantly lower values when the silver pulse was combined with low nutrients. This low nutrient–silver co-exposure-induced decline in photosynthetic pigment concentration may have resulted in additional growth reduction. Photosynthetic pigment content is frequently used as a toxicity indicator for silver in ionic and nanoparticle forms [23,30,31,32]. A reduction in photosynthetic pigment concentration may occur due to ultrastructural changes in chloroplasts and the peroxidation of membrane lipids [33]. The results of this study also indicate that a loss in photosynthetic pigments may be attributed to oxidative stress and damage to the chloroplast membrane, since both nutrient levels and silver pulse treatment resulted in the accumulation of H_2_O_2_ and TBARS (Figure 3). Since ROS function as signal molecules in plants under stress [34], the fast production and accumulation of ROS, such as H_2_O_2_, under nutrient limitation are expected [35,36]. High H_2_O_2_ accumulation was reported for a low availability of nitrogen [35,36], phosphorus [37], and potassium [38,39], with increased activities of NADPH-oxidase and peroxidases being listed as potential sources of ROS [35,39,40]. When the silver pulse treatment co-occurred with low total N and total P levels, the accumulation of H_2_O_2_, as well as the lipid peroxidation process, were more pronounced in comparison to the same silver pulse treatment combined with high nutrient availability (Figure 3). Therefore, our study demonstrated that even very short exposure of *L. gibba* plants to high colloidal silver concentrations resulted in oxidative stress and that total N and P nutrition had a significant effect on the ability of plants to respond to contaminant-induced physiological changes.

Plants can employ several defense mechanisms against ROS and oxidative stress. These defense mechanisms include the activation of antioxidant enzymes, superoxide dismutase, catalase, and peroxidase, as well as accumulating low-molecular-weight antioxidants [41]. The present study showed a decline in non-enzymatic antioxidants, carotenoids, and ascorbic acid in *L. gibba* exposed to a two-hour colloidal silver pulse under both high and low nutrient conditions (Figure 2C and Figure 5). The reduction in carotenoid and ascorbic acid concentration in duckweed induced by metal exposure is well documented [42,43,44]. While the ascorbic acid concentration in *L. gibba* plants was reduced, the activity of APX in colloidal silver-treated plants increased, so we can assume that ascorbic acid is used as an electron donor for the APX-mediated decomposition of H_2_O_2_. In contrast to carotenoids and ascorbic acid, the total soluble phenolic compound concentration was higher in plants exposed to silver under low nutrient conditions (Figure 5B). An increase in total phenolic compounds was reported for other macrophytes exposed to metal toxicity [45,46,47]. Many studies also reported an increase in total phenolic compound concentration in plants exposed to a low availability of nutrients [48,49,50,51,52]. Low nutrient availability enhances the production of carbon-rich secondary metabolites [53]. Since phenolic compounds show a sensitive response to nutrient deficiency prior to the appearance of visible symptoms, Naikoo et al. [54] proposed the assay of phenolic compound concentration as a method for diagnosing nutrient disorders in plants.

Superoxide dismutase represents the first line of defense against oxygen radicals [55], and silver is known to induce SOD activity in plants [33,56,57,58,59]. However, in *L. gibba* plants exposed to colloidal silver pulse, there were no significant differences in the activity of superoxide-dismutase, except for in treatment with the highest silver concentration under high nutrient conditions. Tripathi et al. [60] found that SOD activity in *Scenedesmus* sp. was induced after longer treatment (more than 6 h) with copper and zinc ions. Similar results were reported by Karimi et al. [59] for silver-treated *Triticum aestivum* when the upregulation of SOD occurred only in treatments longer than 6 h. Therefore, two-hour-long pulse exposure of *L. gibba* to colloidal silver may be too short to induce all antioxidative defense mechanisms. In contrast to SOD, the activities of other antioxidative enzymes were induced by the colloidal silver pulse treatment, but to a different extent depending on total N and P nutrition. The low availability of N and P induced the activities of CAT, APX, GPOD, and GR in *Morus alba* plants [61], as well as in *Solanum melongena* plants [62]. Increased activities of antioxidative enzymes may provide better protection from silver-induced oxidative stress. However, the results of this study indicate that the antioxidative response of plants was inadequate to protect the plants from silver-induced oxidative stress. When plants were treated with pulse colloidal silver–low nutrient level co-exposure, significantly higher levels of H_2_O_2_ accumulation and lipid peroxidation were measured in the treated plants, alongside the more pronounced reduction in photosynthetic pigment content in comparison to the same silver pulse treatment combined with high nutrient levels. Rozman and Kalčiková [26] found that changes in environmental conditions may influence the ability of plants to respond to the abiotic stress factor. The results of this study are in agreement with this statement; external nutrient levels had a significant effect on the *L. gibba* plant’s response to colloidal silver presence in the environment. Plants grown and treated under high nutrient levels showed higher free radical scavenging activity which resulted in better overall protection from silver-induced oxidative stress.

Götherberg et al. [63] in a study with water spinach (*Ipomoea aquatica* Forsk.) found that plants accumulated lower concentrations of mercury when metal treatment was combined with higher nutrient concentrations. Similar results were reported for lead accumulation in *L. minor* and *S. polyrhiza* [22]. Therefore, the observed effects of different nutrient levels on *L. gibba* plants treated with colloidal-silver-containing products in this study may be attributed to possibly different concentrations of accumulated silver in *L. gibba* plants under two tested nutrient levels. Future research should be designed to address the limitation of this study and include the metal accumulation at different nutrient levels for a more comprehensive evaluation of the effects of nutrients on the toxicity of different metal pollutants.

In field research, the influences of multiple stress factors are difficult to separate. The impact of multiple stressors depends on the intensity and timing of each stressor [64]. Since nutrient concentrations in the environment are constantly fluctuating, the results of this study should be complemented with data from experiments with a more naturalistic nutrient exposure regime. Additionally, the measurement of the photosynthetic rate may contribute to a better understanding of the long-term consequences that colloidal silver pulse exposure may have on the growth of duckweed plants under different nutrient conditions.

## 4. Materials and Methods

### 4.1. Duckweed Lemna gibba

The duckweed *Lemna gibba* used in this study originated from permanent axenic laboratory cultures maintained in the Department of Biology, University of Osijek (RDSC Clone ID 5597). Plants were cultivated in Erlenmeyer flasks in sterile full-strength Pirson and Seidel’s nutrient solution [65]. The stock cultures were kept in a growth chamber at 25 ± 1 °C under a 16 h photoperiod with the light intensity of 100 µmol photons m^−2^ s^−1^.

### 4.2. Experimental Design

A commercially available suspension of colloidal silver was used for silver treatment. This product was marketed as a sanitizer intended for external use and placed on the market as a colorless liquid in a dark, UV-protective bottle. According to the manufacturer’s label, the product consisted of *Aqua pro injectione* water and silver in a 25 mg/L concentration. A previous study showed that silver in this product is predominantly present in soluble ionic form, but a small portion of silver nanoparticles (average size 95 ± 22.69 nm) was also present [27]. Our earlier study found that this commercially available product stimulated different physiological responses in *L. gibba* grown on the full-strength nutrient solution [27]. Thus, in this study, we focused on the physiological responses in duckweed exposed to colloidal silver suspension in combination with different levels of total nitrogen and phosphorus nutrition. For seven days prior to the colloidal silver pulse exposure, *L. gibba* plants were transferred to modified nutrient solutions set as being of high nutrient concentration (total nitrogen supplied from KNO_3_, TN = 2.2 mmol/L, and total phosphorus supplied from KH_2_PO_4_, TP = 0.73 mmol/L) and low nutrient concentration (TN = 0.44 mmol/L and TP = 0.146 mmol/L). For both tested levels of nutrients, the molar ratio of N to P was 3, the same as in full-strength Pirson and Seidel’s nutrient solution. During this period, the growth of *L. gibba* plants was monitored and the relative growth rate and corresponding doubling time were calculated according to the standard formulas [66]: GR = lnNt_1_-lnNt_0_/t_1_-t_0_, where Nt_0_ represents the number of fronds transferred to the modified nutrient solutions and Nt_1_ represents the total number of fronds after seven days in modified nutrient solutions. The corresponding doubling time was calculated as $T_{d}=\frac{\ln(2)}{GR}$. After seven days, plants grown under both nutrient levels were treated with colloidal silver in nominal concentrations of 100, 200, 500, and 1000 µg/L and nutrient solutions without the addition of colloidal silver were used as controls. A high concentration range for colloidal silver treatment was chosen to represent a pulse toxicity event and plants were exposed to silver pulse for two hours. Therefore, the experiment had a two-way factorial design (nutrient levels and colloidal silver concentration) with the following treatments: I. high nutrient concentration and low nutrient concentration; and II. the colloidal silver treatments Ag_100_, Ag_200_, Ag_500_, and Ag_1000_, as well as the control (0 mg/L of silver). Five replicates were run for each treatment and all experiments were repeated twice. Total plant fresh mass (consisting of healthy-looking duckweed colonies with 3–4 fronds each) added to each replicate Erlenmeyer flask was 600 mg.

### 4.3. Photosynthetic Pigment Content

Samples (0.1 g) of fresh *L. gibba* plants were ground in liquid nitrogen, homogenized in absolute acetone, and then centrifuged at 18,000× *g* and 4 °C for 10 min. Samples were re-extracted until the plant tissue was completely colorless. The extracted solution was used for spectrophotometric measurement of absorption at 644.8, 661.6, and 470 nm. The chlorophyll *a* (Chl *a*), chlorophyll *b* (Chl *b*), and carotenoids (Car) concentrations were calculated according to Lichtenthaler [67] and expressed as mg g^−1^ dry weight.

### 4.4. Biomarkers of Oxidative Stress

Lipid peroxidation was estimated indirectly as the formation of thiobarbituric acid reactive substances (TBARSs) [68]. Approximately 0.2 g of plant tissue per sample was frozen in liquid nitrogen and homogenized with 1 mL of 0.1% trichloroacetic acid (TCA). After centrifugation at 6000× *g* and 4 °C for 5 min, the supernatant (0.5 mL) was combined with 1 mL of 0.5% thiobarbituric acid (TBA) in 20% TCA (*w*/*v*) and incubated at 95 °C for 30 min. The reaction was stopped in an ice bath. The samples were centrifuged at 10,000× *g* and 4 °C for 10 min, and the absorbance was measured at 532 and 600 nm. The amount of TBARS was calculated using the extinction coefficient of 155 mM^−1^ cm^−1^ and expressed as nmol g^−1^ fresh weight.

For H_2_O_2_ content determination, the plant material (approximately 0.2 g per sample) was homogenized in liquid nitrogen, and 1 mL of ice-cold acetone was added. Upon centrifugation at 6000× *g* and 4 °C for 5 min, the supernatant was mixed with 400 μL titanyl-sulfate in acid solution and 500 μL concentrated NH_4_OH. The precipitated complex was dissolved with 1 mL 2 M H_2_SO_4_, and the solution was clarified via centrifugation. The absorbance was read at 415 nm [69] and the H_2_O_2_ concentration was calculated from the calibration curve and expressed as μmol g^−1^ fresh weight.

### 4.5. Antioxidative Response

#### 4.5.1. Protein Extraction and Antioxidant Enzyme Activity Assay

Approximately 0.2 g of fresh plant material per sample was frozen in liquid nitrogen and homogenized in 2 mL potassium phosphate buffer (100 mM, pH 7.0) with the addition of 1 mM EDTA and polyvinylpyrrolidone (PVP). The samples were centrifuged at 18,000× *g* for 15 min at 4 °C. The total soluble protein concentration of the extracts was determined using the Coomassie Brilliant Blue G-250 staining method using bovine serum albumin as a protein standard [70], and results were expressed as μg of protein g^−1^ of fresh weight. The supernatant was also used for the activity assays of antioxidative enzymes.

Superoxide dismutase (SOD, EC 1.15.1.1) activity was assayed using the method of Giannopolitis and Ries [71], determining the inhibition of the photochemical reduction of nitrobluetetrazollium (NBT) in the presence of SOD. The reaction mixture contained 0.05 M KH_2_PO_4_, 0.05 M K_2_HPO_4_, 13 mM methionine, 75 μM NBT, 0.1 mM EDTA, 2 μM riboflavin, and a suitable aliquot of enzyme extract. The test tubes were shaken and placed under a light source for 10 min. The absorbance was measured at 560 nm. The activity of SOD was expressed as unit g^−1^ fresh weight. One unit of SOD was defined as the amount of enzyme needed for 50% inhibition of NBT reduction. Catalase (CAT, EC 1.11.1.6) activity was determined as a decrease in absorbance at 240 nm due to the consumption of H_2_O_2_ following the method of Aebi [72]. The reaction mixture contained 0.05 M KH_2_PO_4_, 0.05 M K_2_HPO_4_, 10 mM of H_2_O_2_ (pH 7.0), and 100 μL of enzyme extract in a total volume of 2 mL. Guaiacol peroxidase (GPOX, EC 1.11.1.7) activity was determined by the method of Siegel and Galston [73], as an increase in absorbance at 470 nm due to guaiacol polymerizing to tetraguaiacol. The reaction mixture contained 5 mM guaiacol, 0.2 M KH_2_PO_4_, 0.2 M Na_2_HPO_4_ × 12 H_2_O, 5 mM H_2_O_2_, and 200 μL of enzyme extract in 2 mL of the total volume of the reaction mixture. Ascorbate peroxidase (APX, EC 1.11.1.11) was determined following the protocol of Nakano and Asada [74]. The reaction mixture contained 0.05 M KH_2_PO_4_, 0.05 M K_2_HPO_4_, 0.1 mM EDTA, 5 mM ascorbic acid, 12 mM of H_2_O_2_, and 180 μL of protein extract in a total volume of 2 mL. Glutathione reductase (GR, EC 1.6.4.2) activity was assayed according to Dolphin et al. [75]. The reaction mixture contained 0.1 M KH_2_PO_4_, 0.1 M K_2_HPO_4_, 1 mM EDTA, 2 mM NADPH, 2 mM GSSG, and 50 μL of protein extract in 2 mL of total volume. The activity of glutathione S-transferase (GST, EC 2.5.1.18) was determined by the method of Habig et al. [76] using 1-chloro-2,4-dinitrobenzene (CDNB) as the substrate. The reaction mixture contained 0.1 M KH_2_PO_4_, 0.1 M K_2_HPO_4_, 1 mM EDTA, 75 mM reduced glutathione, 30 mM CDNB, and 100 μL of enzyme extract, and the absorbance was measured at 340 nm.

#### 4.5.2. Ascorbic Acid Concentration, Soluble Phenolic Compound Concentration, and Free Radical Scavenging Activity

The ascorbic acid concentration was determined spectrophotometrically [69]. Plant material (approximately 0.05 g per sample) was homogenized in 1 mL of 6% thiobarbituric acid (TBA), 0.5 mL of 2% dinitrophenylhydrazine (DNPH), and 10% thiourea in 70% ethanol. Samples were then incubated in boiling water for 15 min, cooled to ambient temperature, and centrifuged at 6000× *g* at 4 °C for 10 min. After adding 1 mL of 80% (*w*/*v*) H_2_SO_4_ in an ice bath, the absorbance of the mixture was measured at 530 nm, and the concentration of ascorbate was calculated using the extinction coefficient of ε = 226.2 mM^−1^ cm^−1^. The concentration of ascorbate was expressed as μmol g^−1^ fresh weight.

Total soluble phenolic compound concentration was determined spectrophotometrically following the Folin–Ciocalteu method [77]. Approximately 0.1 g of fresh plant tissue per sample was frozen in liquid nitrogen and extracted with 1 mL of 80% ethanol for 24 h at −20 °C. After extraction, samples were centrifuged at 15,000× *g* at 4 °C for 10 min. The reaction mixture contained 100 μL of plant alcohol extract, 700 μL of H_2_O, 100 μL of Folin–Ciocalteu reagent, and 150 μL of the saturated Na_2_CO_3_ solution. The absorbance was measured at 765 nm, and the concentration of phenolic compounds was calculated from the calibration curve of known amounts of gallic acid and expressed as mg gallic acid equivalent (GAE) g^−1^ fresh weight.

Free radical scavenging activity was determined according to the DPPH method [78]. Samples (0.1 g) were extracted with 1 mL of 80% ethanol for 24 h at −20 °C. After extraction, samples were centrifuged at 15,000× *g* and 4 °C for 10 min. The reaction mixture contained 20 μL of plant alcohol extract and 980 μL of DPPH (1,1-diphenyl-2-picrylhydrazyl) solution. Free radical scavenging activity was determined from a calibration curve of known amounts of Trolox (6-hydroxy-2,5,7,8-tetramethylchroman-2-carboxylic acid) and expressed as μmol_Trolox_ g^−1^ fresh weight.

All chemicals and reagents were purchased from Sigma-Aldrich (Steinheim, Germany).

### 4.6. Data Analysis

Values shown in the figures and tables are mean values ± standard deviation of ten replicates (*n* = 10). Every replicate represents a sample from a separate Erlenmeyer flask. The paired sample *t*-test was performed to analyze the significant differences in growth parameters between the high nutrient and low nutrient groups. Significant effects of different nutrient levels combined with pulse colloidal silver treatment were tested with factorial analysis of variance followed by the Tukey HSD post hoc test. Differences among groups were considered to be statistically significant at *p* < 0.05. Data transformations were applied to approximate the assumptions of normality and the same error variance.

## 5. Conclusions

This study revealed that the external concentrations of nitrogen and phosphorus in the nutrient solution are important for *L. gibba*’s ability to respond to short-term treatment with the colloidal-silver-containing product as a potential pollutant in the aquatic environment. Short-term pulse exposure to colloidal silver product resulted in oxidative stress in *L. gibba* plants under both high and low nutrient levels. Oxidative stress was evident due to the accumulation of hydrogen peroxide and lipid peroxidation products, as well as reduced concentrations of photosynthetic pigments. However, when plants were treated with the colloidal silver product under high nutrient levels, observed oxidative stress was less pronounced compared to plants treated under low nutrient levels. Although plants treated under low nutrient levels had higher activities of antioxidative enzymes, such as CAT and GR, and higher concentrations of phenolic compounds, their antioxidative response was inadequate to protect the plants from the toxic effects of the applied colloidal silver product. Our results suggest that high nitrogen and phosphorus concentrations improved the tolerance of *L. gibba* to a commercially available product containing colloidal silver. The production and consumption of colloidal silver products are increasing, and this commercially available source of silver poses a severe threat to primary producers in aquatic environments.

## Figures and Tables

**Table 1:** The growth rate of *L. gibba* plants after a seven-day cultivation period in nutrient solutions with high and low total nitrogen and total phosphorus concentrations. Values shown are mean ± standard deviation of ten replicates (*n* = 10). Different letters indicate significantly different values (*t*-test, *p* ≤ 0.05).

	Frond Number Growth Rate (Day^−1^)	Doubling Time (Day)	Fresh Weight Growth Rate (Day^−1^)
High nutrient	0.29 ± 0.03 ^a^	2.40 ± 0.19 ^a^	0.25 ± 0.03 ^a^
Low nutrient	0.18 ± 0.02 ^b^	3.81 ± 0.45 ^b^	0.16 ± 0.02 ^b^

## Data Availability

The data presented in this study are available upon request from the corresponding author.

## References

[1] Ceschin S., Crescenzi M., Iannelli M.A. (2020). Phytoremediation Potential of the Duckweeds *Lemna minuta* and *Lemna minor* to Remove Nutrients from Treated Waters. Environ. Sci. Pollut. Res..

[2] Häder D.P., Banaszak A.T., Villafañe V.E., Narvarte M.A., González R.A., Helbling E.W. (2020). Anthropogenic Pollution of Aquatic Ecosystems: Emerging Problems with Global Implications. Sci. Total Environ..

[3] Bashir I., Lone F.A., Bhat R.A., Mir S.A., Dar Z.A., Dar S.A. (2020). Concerns and Threats of Contamination on Aquatic Ecosystems. Bioremediation and Biotechnology: Sustainable Approaches to Pollution Degradation.

[4] Hampel M., Blasco J., Segner H. (2015). Molecular and Cellular Effects of Contamination in Aquatic Ecosystems. Environ. Sci. Pollut. Res..

[5] Fulton B.A., Brain R.A., Usenko S., Back J.A., King R.S., Brooks B.W. (2009). Influence of Nitrogen and Phosphorus Concentrations and Ratios on *Lemna gibba* Growth Responses to Triclosan in Laboratory and Stream Mesocosm Experiments. Environ. Toxicol. Chem..

[6] Rogers K.R., Navratilova J., Stefaniak A., Bowers L., Knepp A.K., Al-Abed S.R., Potter P., Gitipour A., Radwan I., Nelson C. (2018). Characterization of Engineered Nanoparticles in Commercially Available Spray Disinfectant Products Advertised to Contain Colloidal Silver. Sci. Total Environ..

[7] Cascio C., Geiss O., Franchini F., Ojea-Jimenez I., Rossi F., Gilliland D., Calzolai L. (2015). Detection, Quantification and Derivation of Number Size Distribution of Silver Nanoparticles in Antimicrobial Consumer Products. J. Anal. At. Spectrom..

[8] Lim J.-H., Bairi V.G., Linder S.W., Fong A. (2019). Detection and Characterization of Silver Nanostructures in Consumer Products. J. Nanosci. Nanotechnol..

[9] Wasukan N., Srisung S., Kulthong K., Boonrungsiman S., Maniratanachote R. (2015). Determination of Silver in Personal Care Nanoproducts and Effects on Dermal Exposure. J. Nanoparticle Res..

[10] Zhang W., Xiao B., Fang T. (2018). Chemical Transformation of Silver Nanoparticles in Aquatic Environments: Mechanism, Morphology and Toxicity. Chemosphere.

[11] Gottschalk F., Sonderer T., Scholz R.W., Nowack B. (2009). Modeled Environmental Concentrations of Engineered Nanomaterials (TiO_2_, ZnO, Ag, CNT, Fullerenes) for Different Regions. Environ. Sci. Technol..

[12] Zhang W., Ke S., Sun C., Xu X., Chen J., Yao L. (2019). Fate and Toxicity of Silver Nanoparticles in Freshwater from Laboratory to Realistic Environments: A Review. Environ. Sci. Pollut. Res..

[13] Pem B., Ćurlin M., Jurašin D.D., Vrček V., Barbir R., Micek V., Fratila R.M., de la Fuente J.M., Vrček I.V. (2021). Fate and Transformation of Silver Nanoparticles in Different Biological Conditions. Beilstein J. Nanotechnol..

[14] Angel B.M., Goodwyn K., Jolley D.F., Simpson S.L. (2018). The Use of Time-Averaged Concentrations of Metals to Predict the Toxicity of Pulsed Complex Effluent Exposures to a Freshwater Alga. Environ. Pollut..

[15] Angel B.M., Simpson S.L., Granger E., Goodwyn K., Jolley D.F. (2017). Time-Averaged Concentrations Are Effective for Predicting Chronic Toxicity of Varying Copper Pulse Exposures for Two Freshwater Green Algae Species. Environ. Pollut..

[16] Belgers J.D.M., Aalderink G.H., Arts G.H.P., Brock T.C.M. (2011). Can Time-Weighted Average Concentrations Be Used to Assess the Risks of Metsulfuron-Methyl to *Myriophyllum spicatum* under Different Time-Variable Exposure Regimes?. Chemosphere.

[17] Razinger J., Dermastia M., Drinovec L., Drobne D., Zrimec A., Dolenc Koce J. (2007). Antioxidative Responses of Duckweed (*Lemna minor* L.) to Short-Term Copper Exposure. Environ. Sci. Pollut. Res. Int..

[18] Begović L., Mlinarić S., Antunović Dunić J., Katanić Z., Lončarić Z., Lepeduš H., Cesar V. (2016). Response of *Lemna minor* L. to Short-Term Cobalt Exposure: The Effect on Photosynthetic Electron Transport Chain and Induction of Oxidative Damage. Aquat. Toxicol..

[19] Razinger J., Dermastia M., Koce J.D., Zrimec A. (2008). Oxidative Stress in Duckweed (*Lemna minor* L.) Caused by Short-Term Cadmium Exposure. Environ. Pollut..

[20] Roubeau Dumont E., Larue C., Pujol B., Lamaze T., Elger A. (2019). Environmental Variations Mediate Duckweed (*Lemna minor* L.) Sensitivity to Copper Exposure through Phenotypic Plasticity. Environ. Sci. Pollut. Res..

[21] Yang J., Li G., Xia M., Chen Y., Chen Y., Kumar S., Sun Z., Li X., Zhao X., Hou H. (2022). Combined Effects of Temperature and Nutrients on the Toxicity of Cadmium in Duckweed (*Lemna aequinoctialis*). J. Hazard. Mater..

[22] Leblebici Z., Aksoy A. (2011). Growth and Lead Accumulation Capacity of Lemna Minor and *Spirodela polyrhiza* (*Lemnaceae*): Interactions with Nutrient Enrichment. Water Air Soil Pollut..

[23] Yu H., Qi W., Cao X., Wang Y., Li Y., Xu Y., Zhang X., Peng J., Qu J. (2022). Impact of Microplastics on the Foraging, Photosynthesis and Digestive Systems of Submerged Carnivorous Macrophytes under Low and High Nutrient Concentrations. Environ. Pollut..

[24] Lemon G.D., Posluszny U., Husband B.C. (2001). Potential and Realized Rates of Vegetative Reproduction in *Spirodela polyrhiza*, *Lemna minor*, and *Wolffia borealis*. Aquat. Bot..

[25] van Hoeck A., Horemans N., Monsieurs P., Cao H.X., Vandenhove H., Blust R. (2015). The First Draft Genome of the Aquatic Model Plant *Lemna minor* Opens the Route for Future Stress Physiology Research and Biotechnological Applications. Biotechnol. Biofuels.

[26] Rozman U., Kalčíková G. (2022). The Response of Duckweed *Lemna minor* to Microplastics and Its Potential Use as a Bioindicator of Microplastic Pollution. Plants.

[27] Varga M., Horvatić J., Barišić L., Lončarić Z., Dutour Sikirić M., Erceg I., Kočić A., Štolfa Čamagajevac I. (2019). Physiological and Biochemical Effect of Silver on the Aquatic Plant *Lemna gibba* L.: Evaluation of Commercially Available Product Containing Colloidal Silver. Aquat. Toxicol..

[28] Tripathi A., Liu S., Singh P.K., Kumar N., Pandey A.C., Tripathi D.K., Chauhan D.K., Sahi S. (2017). Differential Phytotoxic Responses of Silver Nitrate (AgNO_3_) and Silver Nanoparticle (AgNPs) in *Cucumis sativus* L.. Plant Gene.

[29] la Point T.W., Waller W.T. (2000). Field Assessments in Conjunction with Whole Effluent Toxicity Testing. Environ. Toxicol. Chem..

[30] Hazeem L.J., Kuku G., Dewailly E., Slomianny C., Barras A., Hamdi A., Boukherroub R., Culha M., Bououdina M. (2019). Toxicity Effect of Silver Nanoparticles on Photosynthetic Pigment Content, Growth, ROS Production and Ultrastructural Changes of Microalgae *Chlorella vulgaris*. Nanomaterials.

[31] Jiang H.S., Li M., Chang F.Y., Li W., Yin L.Y. (2012). Physiological Analysis of Silver Nanoparticles and AgNO_3_ Toxicity to *Spirodela polyrhiza*. Environ. Toxicol. Chem..

[32] Ksiązyk M., Asztemborska M., Stęborowski R., Bystrzejewska-Piotrowska G. (2015). Toxic Effect of Silver and Platinum Nanoparticles toward the Freshwater Microalga *Pseudokirchneriella subcapitata*. Bull. Environ. Contam. Toxicol..

[33] Xu Q.S., Hu J.Z., Xie K.B., Yang H.Y., Du K.H., Shi G.X. (2010). Accumulation and Acute Toxicity of Silver in *Potamogeton crispus* L.. J. Hazard. Mater..

[34] Quan L.J., Zhang B., Shi W.W., Li H.Y. (2008). Hydrogen Peroxide in Plants: A Versatile Molecule of the Reactive Oxygen Species Network. J. Integr. Plant Biol..

[35] Shin R., Schachtman D.P. (2004). Hydrogen Peroxide Mediates Plant Root Cell Response to Nutrient Deprivation. Proc. Natl. Acad. Sci. USA.

[36] Shin R., Berg R.H., Schachtman D.P. (2005). Reactive Oxygen Species and Root Hairs in *Arabidopsis* Root Response to Nitrogen, Phosphorus and Potassium Deficiency. Plant Cell Physiol..

[37] Malusà E., Laurenti E., Juszczuk I., Ferrari R.P., Rychter A.M. (2002). Free Radical Production in Roots of *Phaseolus vulgaris* Subjected to Phosphate Deficiency Stress. Plant Physiol. Biochem..

[38] Hernandez M., Fernandez-Garcia N., Garcia-Garma J., Rubio-Asensio J.S., Rubio F., Olmos E. (2012). Potassium Starvation Induces Oxidative Stress in *Solanum lycopersicum* L. Roots. J. Plant Physiol..

[39] Kim M.J., Ciani S., Schachtman D.P. (2010). A Peroxidase Contributes to ROS Production during *Arabidopsis* Root Response to Potassium Deficiency. Mol. Plant.

[40] Ho C.H., Tsay Y.F. (2010). Nitrate, Ammonium, and Potassium Sensing and Signaling. Curr. Opin. Plant Biol..

[41] Štolfa I., Pfeiffer T.Ž., Špoljarić D., Teklić T., Lončarić Z. (2015). Heavy Metal-Induced Oxidative Stress in Plants: Response of the Antioxidative System. Reactive Oxygen Species and Oxidative Damage in Plants Under Stress.

[42] Hou W., Chen X., Song G., Wang Q., Chi Chang C. (2007). Effects of Copper and Cadmium on Heavy Metal Polluted Waterbody Restoration by Duckweed (*Lemna minor*). Plant Physiol. Biochem..

[43] Radić S., Stipaničev D., Cvjetko P., Mikelić I.L., Rajčić M.M., Širac S., Pevalek-Kozlina B., Pavlica M. (2010). Ecotoxicological Assessment of Industrial Effluent Using Duckweed (*Lemna minor* L.) as a Test Organism. Ecotoxicology.

[44] Xing W., Li D., Liu G. (2010). Antioxidative Responses of *Elodea nuttallii* (Planch.) H. St. John to Short-Term Iron Exposure. Plant Physiol. Biochem..

[45] Sivaci A., Sivaci E.R., Sökmen M. (2007). Changes in Antioxidant Activity, Total Phenolic and Abscisic Acid Constituents in the Aquatic Plants *Myriophyllum spicatum* L. and *Myriophyllum triphyllum* Orchard Exposed to Cadmium. Ecotoxicology.

[46] Bizzo A.L.T., Intorne A.C., Gomes P.H., Suzuki M.S., Esteves B.D.S. (2014). Short-Term Physiological Responses to Copper Stress in *Salvinia auriculata* Aubl. Acta Limnol. Bras..

[47] Krishnaraj C., Jagan E.G., Ramachandran R., Abirami S.M., Mohan N., Kalaichelvan P.T. (2012). Effect of Biologically Synthesized Silver Nanoparticles on *Bacopa monnieri* (Linn.) Wettst. Plant Growth Metabolism. Process Biochem..

[48] Lillo C. (2008). Signalling Cascades Integrating Light-Enhanced Nitrate Metabolism. Biochem. J..

[49] Urbanczyk-Wochniak E., Fernie A.R. (2005). Metabolic Profiling Reveals Altered Nitrogen Nutrient Regimes Have Diverse Effects on the Metabolism of Hydroponically-Grown Tomato (*Solanum lycopersicum*) Plants. J. Exp. Bot..

[50] Le Bot J., Bénard C., Robin C., Bourgaud F., Adamowicz S. (2009). The “trade-off” between Synthesis of Primary and Secondary Compounds in Young Tomato Leaves is Altered by Nitrate Nutrition: Experimental Evidence and Model Consistency. J. Exp. Bot..

[51] Løvdal T., Olsen K.M., Slimestad R., Verheul M., Lillo C. (2010). Synergetic Effects of Nitrogen Depletion, Temperature, and Light on the Content of Phenolic Compounds and Gene Expression in Leaves of Tomato. Phytochemistry.

[52] Nguyen P.M., Niemeyer E.D. (2008). Effects of Nitrogen Fertilization on the Phenolic Composition and Antioxidant Properties of Basil (*Ocimum basilicum* L.). J. Agric. Food Chem..

[53] Stewart A.J., Chapman W., Jenkins G.I., Graham I., Martin T., Crozier A. (2001). The Effect of Nitrogen and Phosphorus Deficiency on Flavonol Accumulation in Plant Tissues. Plant Cell Environ..

[54] Naikoo M.I., Dar M.I., Raghib F., Jaleel H., Ahmad B., Raina A., Khan F.A., Naushin F. (2019). Role and Regulation of Plants Phenolics in Abiotic Stress Tolerance: An Overview. Plant Signaling Molecules: Role and Regulation Under Stressful Environments.

[55] Khataee A.R., Movafeghi A., Torbati S., Salehi Lisar S.Y., Zarei M. (2012). Phytoremediation Potential of Duckweed (*Lemna minor* L.) in Degradation of C.I. Acid Blue 92: Artificial Neural Network Modeling. Ecotoxicol. Environ. Saf..

[56] Hu C., Liu Y., Li X., Li M. (2013). Biochemical Responses of Duckweed (*Spirodela polyrhiza*) to Zinc Oxide Nanoparticles. Arch. Environ. Contam. Toxicol..

[57] Jiang H.S., Qiu X.N., Li G.B., Li W., Yin L.Y. (2014). Silver Nanoparticles Induced Accumulation of Reactive Oxygen Species and Alteration of Antioxidant Systems in the Aquatic Plant *Spirodela polyrhiza*. Environ. Toxicol. Chem..

[58] Yuan L., Richardson C., Ho M., Willis C.W., Colman B.P., Wiesner M.R. (2018). Stress Responses of Aquatic Plants to Silver Nanoparticles. Environ. Sci. Technol..

[59] Karimi J., Mohsenzadeh S., Niazi A., Moghadam A. (2017). Differential Expression of Mitochondrial Manganese Superoxide Dismutase (SOD) in *Triticum aestivum* Exposed to Silver Nitrate and Silver Nanoparticles. Iran. J. Biotechnol..

[60] Tripathi B.N., Mehta S.K., Amar A., Gaur J.P. (2006). Oxidative Stress in *Scenedesmus* sp. during Short- and Long-Term Exposure to Cu^2+^ and Zn^2+^. Chemosphere.

[61] Tewari R.K., Kumar P., Sharma P.N. (2007). Oxidative Stress and Antioxidant Responses in Young Leaves of Mulberry Plants Grown under Nitrogen, Phosphorus or Potassium Deficiency. J. Integr. Plant Biol..

[62] Singh M., Singh V.P., Prasad S.M. (2016). Nitrogen Modifies NaCl Toxicity in Eggplant Seedlings: Assessment of Chlorophyll a Fluorescence, Antioxidative Response and Proline Metabolism. Biocatal. Agric. Biotechnol..

[63] Göthberg A., Greger M., Holm K., Bengtsson B.-E. (2004). Influence of Nutrient Levels on Uptake and Effects of Mercury, Cadmium, and Lead in Water Spinach. J. Environ. Qual..

[64] Gunderson A.R., Armstrong E.J., Stillman J.H. (2016). Multiple Stressors in a Changing World: The Need for an Improved Perspective on Physiological Responses to the Dynamic Marine Environment. Annu. Rev. Mar. Sci..

[65] Pirson A., Seidel F. (1950). Cell Metabolism and Physiology in *Lemna minor* Root Deprived of Potassium and Calcium (Zell- Und Stoffwechselphysiologiche Untersuchungen an Der Wurzel von *Lemna minor* Unter Besonderer Berücksichtigung von Kalium Und Calciummangel). Planta.

[66] OECD (2006). Test No. 221: *Lemna* sp. Growth Inhibition Test. OECD Guidelines for the Testing of Chemicals, Section 2.

[67] Lichtenthaler H.K. (1987). Chlorophylls and Carotenoids: Pigments of Photosynthetic Biomembranes. Methods Enzymol..

[68] Verma S., Dubey R.S. (2003). Lead Toxicity Induces Lipid Peroxidation and Alters the Activities of Antioxidant Enzymes in Growing Rice Plants. Plant Sci..

[69] Mukherjee S.P., Choudhuri M.A. (1983). Implications of Water Stress-Induced Changes in the Levels of Endogenous Ascorbic Acid and Hydrogen Peroxide in *Vigna* Seedlings. Physiol. Plant..

[70] Bradford M.M. (1976). A Rapid and Sensitive Method for the Quantitation of Microgram Quantities of Protein Utilizing the Principle of Protein-Dye Binding. Anal. Biochem..

[71] Giannopolitis C.N., Ries S.K. (1977). Superoxide Dismutases: I. Occurrence in Higher Plants. Plant Physiol..

[72] Aebi H. (1984). Catalase in *Vitro*. Methods in Enzymology.

[73] Siegel B.Z., Galston A.W. (1967). The Isoperoxidases of *Pisum sativum*. Plant Physiol..

[74] Nakano Y., Asada K. (1981). Hydrogen Peroxide Is Scavenged by Ascorbate-Specific Peroxidase in Spinach Chloroplasts. Plant Cell Physiol..

[75] Dolphin D., Avramović O., Poulson R. (1989). Glutathione: Chemical, Biochemical, and Medical Aspects.

[76] Habig W.H., Pabst M.J., Jakoby W.B. (1974). Glutathione S-Transferases. The First Enzymatic Step in Mercapturic Acid Formation. J. Biol. Chem..

[77] Singleton V.L., Rossi J.A. (1965). Colorimetry of Total Phenolics with Phosphomomoybdic-Phosphotungstic Acid Reagents. Am. J. Enol. Viticult..

[78] Brand-Williams W., Cuvelier M.E., Berset C. (1995). Use of a Free Radical Method to Evaluate Antioxidant Activity. LWT Food Sci. Technol..

